# A multicenter registry of neuropsychiatric outpatients in Korean medicine hospitals (KMental): Protocol of a prospective, multicenter, registry study

**DOI:** 10.1097/MD.0000000000032151

**Published:** 2022-12-09

**Authors:** Hyo-Weon Suh, Youme Ko, Seunghwan Moon, Jong Woo Kim, Sun-Yong Chung, Sunggyu Hong, Hyun Woo Lee, Ching-Wen Huang, Bo-Hyoung Jang

**Affiliations:** a Health Policy Research Team, Division of Healthcare Research, National Evidence-based Healthcare Collaborating Agency, Gwangjin-gu, Seoul, Republic of Korea; b Department of Preventive Medicine, College of Korean Medicine, Kyung Hee University, Dongdaemoon-gu, Seoul, Republic of Korea; c Institute of Safety and Efficacy, Effectiveness Evaluation for Korean Medicine, Dongdaemoon-gu, Seoul, Republic of Korea; d Department of Global Public Health and Korean Medicine Management, Graduate School, Kyung Hee University, Seoul, South Korea; e Department of Clinical Korean Medicine, Graduate School, Kyung Hee University, Dongdaemoon-gu, Seoul, Republic of Korea; f Department of Neuropsychiatry, Kyung Hee University Korean Medicine Hospital at Gangdong, Gangdong-gu, Seoul, Republic of Korea.

**Keywords:** complementary therapies, cost of illness, Korean traditional medicine, mental health, registries

## Abstract

Mental health is an important global health concern. In Korea, many people visit Korean medicine clinics due to Stagnation syndrome and Hwabyung (cultural idioms of distress).

**Methods and analysis:**

We established a research collaboration comprising of professionals from 8 Korean medicine hospitals and planned a prospective, multicenter registry study. Demographic, socioeconomic, and clinical data will be collected from patients with mental disorders visiting the Korean medicine neuropsychiatric department of Korean medicine hospitals. We will include major psychiatric diagnoses related to neurosis and Korean traditional mental disorders such as Stagnation syndrome and Hwabyung.

We expect to identify comprehensive characteristics of participants, measure the severity of their symptoms or distress, and investigate patterns of Korean medicine interventions and treatment responses for both the short-term and long-term (at least 4 years). Additionally, this study will include Health Insurance Review & Assessment Service data to analyze the medical use of registered patients before and after registration, in case the participant submits the consent form for personal information collection and use.

To analyze the effectiveness of Korean medicine treatment for the participants, stratified or corrected analyses of age, sex, and diagnosis will be performed. The difference in the change in the psychometric measurements, quality of life measured by short form 36, and quality-adjusted life years will be calculated to evaluate the effectiveness of Korean medicine treatment.

## 1. Introduction

Mental disorders are risk factors for all-cause mortality. Incidents of death because of suicide and self-injury are 7.22-times more in mental health patients than in the general population; approximately eight million annual deaths worldwide—1/7 of the total deaths—in 2012 are attributable to mental disorders.^[1]^ This makes mental health a global concern.

In Korea, the mental health budget in 2019 was USD 253.4 million.^[2]^ A survey revealed that in Korea the lifetime prevalence of depressive and anxiety disorders was 7.7% and 9.3%, respectively—their annual prevalence was 1.7% and 3.1%, respectively, in 2021.^[3]^ The prevalence of all mental disorders tended to be higher in the survey than in the registry in 2006 (15.2% vs 7.83%), 2011 (15.4% vs 9.60%), and 2016 (12.8% vs 11.63%), implying a treatment gap.^[4]^ Although the same study reported that the registry-based prevalence of major depressive disorder (MDD) exceeded the survey-based prevalence of MDD in 2016 (3.41% vs 1.6%) and suggested a change in perspectives toward mental disorders,^[4]^ there is still stigma attached to mental disorders in Korea.

Many researchers have analyzed the stigma of mental illness among Koreans and Korean immigrants and the role of culture in this issue.^[5–8]^ In this cultural context, Koreans with psychological distress visit clinics of Korean medicine as an alternative.

The Korean medicine system considers psychological distress as a response to, or a transient result from, stressful daily events, and not as disorders that imply “abnormality” or “immaturity.” Stagnation syndrome ^[9]^ and Hwabyung ^[10]^—two mental disorders reflective of the East Asian culture—were recently introduced in an international journal by Chinese and Korean researchers, respectively. This represents the starting point from where various features of traditional mental disorders can be academically discussed worldwide.

Stagnation syndrome is similar to MDD and is defined as “a general term for diseased states characterized by a depressed mood with feelings of despair or uneasiness” in the *International Standard Terminologies on Traditional Medicine* of the World Health Organization.^[11]^ The prevalence of Stagnation syndrome was 6.2% in community samples from Hong Kong.^[12]^

Hwabyung is described as “a Korean folk syndrome literally translated into English as ‘anger syndrome,’ and is attributed to the suppression of anger. The symptoms include insomnia, fatigue, panic, fear of impending death, dysphoric affect, indigestion, anorexia, dyspnea, palpitations, generalized aches and pains, and a feeling of a mass in the epigastrium.” in Appendix I of the Diagnostic and Statistical Manual of Mental Disorders, 4^th^ edition (DSM-IV).^[13]^ Hwabyung is listed in the Korean Standard Classification of Diseases 7^th^ Revision as U22.2, which is one of the U codes (codes for special purposes). According to the Korean Health Insurance Review & Assessment Service data, the number of patients who used healthcare services to treat hwabyung was 12,131 in 2020. The prevalence of Hwabyung has been reported to range from 4.2% to 13.3% in Korean community samples.^[14–17]^ A clinical practice guideline for traditional Korean medicine has been developed because of the high prevalence of Hwabyung patients and their frequent clinic visits.^[18]^

We established a research collaboration (named “Korean Medicine Mental Health”) which consisted of professionals in Korean medicine hospitals and planned a prospective observational study to understand how Korean medicine manages mental disorders and psychological distress, explore patients’ perceptions and anticipations, and evaluate results of Korean medicine treatment. In particular, we will pay attention to Stagnation syndrome and Hwabyung. This study is expected to improve our understanding of traditional mental disorders.

### 1.1. Study objectives

The objectives of the study are:

- To develop a multicenter registry for mental disorders, including diagnostic and statistical manual of mental disorders (DSM) disorders and traditional mental disorders such as Stagnation syndrome and Hwabyung in Korea.
- To describe the demographic and socioeconomic characteristics, comorbidities, and symptom severity of patients who visited neuropsychiatric outpatients in a Korean medicine hospital.
- To explore patients’ perceptions toward the reasons they are suffering from psychological distress.
- To investigate the details of Korean medicine interventions to determine core interventions for mental disorders.
- To evaluate short-term treatment response, to observe the prognosis of mental disorders or distress, and to incorporate baseline data with regular follow-up data for at least 4 years.
- To analyze the medical use of Korean medicine and Western medicine in patients.
- To facilitate attention and interest on Stagnation syndrome and Hwabyung in general psychiatry.

## 2. Methods and analysis

### 2.1. Study design

This study—“KMental” for short—is a multicenter, prospective, registry study of adult patients with mental disorders in Korean medicine hospitals. The institutional review board (IRB) of the Kyung Hee University Korean Medicine Hospital at Gangdong approved this study (KHNMCOH 2021-04-003). The version 1.0 was approved on 27 April 2021. Lastly, update version 4.0 was approved on 19 May 2022.

Data will be collected from patients with mental disorders visiting the Korean medicine neuropsychiatric department to identify sociodemographic characteristics, investigate psychiatric distress, and analyze patterns of Korean medicine interventions and treatment responses. Additionally, we will compare and analyze the medical use of registered patients before and after registration based on the Health Insurance Review & Assessment Service data if they consent to the use of personal information.

This study will be conducted at the following 8 Korean medicine hospitals: the Kyung Hee University Korean Medicine Hospital at Gangdong; the Dongshin University Gwangju Oriental Hospital; the Pohang Korean Medicine Hospital of Daegu Haany University; the Dong-Eui University Oriental Medicine Hospital; the Pusan National University Korean Medicine Hospital; the Sangji University Korean Medical Center; the Chungju Oriental Hospital of Semyung University; and the Woosuk University Korean Medicine Medical Center. Approval was received from the IRBs of all participating institutions. In case more hospitals want to participate in the KMental Registry in the future, the number of hospitals can be increased.

### 2.2. Study participants

#### 2.2.1. Diagnostic criteria.

Mental disorders will be diagnosed based on the DSM-5.^[19]^ Eligibility for this study will be assessed based on the structured clinical interview for DSM-5—clinician version.^[20]^ According to the structured clinical interview for DSM-5, mental disorders are diagnosed as follows: depressive disorder, panic disorder, generalized anxiety disorder, acute stress disorder, post-traumatic stress disorder, and adjustment disorder. We will also diagnose somatic symptom and insomnia disorders according to the DSM-5.

Additionally, we will focus on traditional mental disorders, especially Stagnation syndrome and Hwabyung. These are listed as “Depression disorder” and “Repressed fire disorder,” respectively, in the Traditional Medicine chapter of the international statistical classification of diseases and related health problems, 11^th^ revision.^[21]^ However, “Stagnation syndrome” and “Hwabyung” were more actively used in academic articles (see previous articles^[9,22,23]^ and,^[24–27]^ respectively). Thus, we prefer to use this terminology in this article.

Stagnation syndrome will be diagnosed according to the *Criteria of Diagnosis and Therapeutic Effect of Diseases and Syndromes in Traditional Chinese Medicine* (ZY/T001.1-94).^[28]^ We translated these criteria from Chinese into Korean.

Hwabyung will be diagnosed using the Hwa-Byung Diagnostic Interview Schedule.^[29]^ These criteria have been validated and widely used in research on Hwabyung, Korea.^[10]^

#### 2.2.2. Inclusion criteria.

1. Adults aged 20 to 65 years.
2. A person diagnosed with at least one of the following diagnostic codes: depressive disorder (F32 or F33), panic disorder (F41.0), generalized anxiety disorder (F41.1), acute stress disorder (F43.0), post-traumatic stress disorder (F43.1), adjustment disorder (F43.2), somatic symptom disorder (F45.1), insomnia disorder (F51.1), Stagnation syndrome (U22.1), or Hwabyung (U22.2).

#### 2.2.3. Exclusion criteria.

1. Severe mental illness such as schizophrenia or delusional disorder.
2. Neurological disorders such as major cognitive impairment (dementia) or epilepsy, intellectual disability, or personality disorders.
3. Seriously unstable medical conditions (e.g., comorbidities with cancer, cerebral vascular disease, chronic liver diseases, chronic renal disease, etc.)
4. A condition in which it is difficult for the participant to perform the interview and questionnaire tests conducted in this study (e.g., if there is difficulty in reading, writing, listening, speaking, or understanding)

#### 2.2.4. Withdrawal criteria.

1. The participant or guardian has expressed intention to withdraw the study.
2. Loss of follow-up (e.g., contact with the participant is not possible for a long time)
3. Impossible to proceed with the study because of an event that occurs to the participant during the study.

### 2.3. Recruitment

In the Kyung Hee University Korean Medicine Hospital at Gangdong, participants were recruited through advertising posters displayed in hospitals from January 2022; the first participant was registered on 15 March, 2022. The other sites will identify and recruit participants at outpatient clinics after receiving approval from their respective IRBs.

### 2.4. Intervention

Management of mental disorders will be performed at the discretion of doctors of Korean medicine (majoring in neuropsychiatry) at each registry site. We did not intend to standardize the management of all participating practitioners.

### 2.5. Outcome measures

If a participant has voluntarily signed the consent form and is eligible according to the inclusion criteria, a research registration number will be assigned, and the participant’s baseline investigation will be conducted. Thereafter, information about interventions and patients’ clinical courses will be collected during treatment for up to 3 months, and a total of 5 follow-up visits will be independently conducted (i.e., half-yearly and annual visits after registration) (see Table 1 for an overview).

**Table 1:** Overview of the assessment time points.

	Study Period							
	Screening	Registration	Follow-up					
TIMEPOINT**	S (baseline)	V1 (baseline)	Unscheduled visits (upto 3 mo)	V2 (6 mo)	V3 (1 yr)	V4 (2 yrs)	V5 (3 yrs)	V6 (4 yrs)
**ENROLMENT:**								
Eligibility screen	X							
Informed consent	X							
**INTERVENTIONS:**								
		Unscheduled visits						
**ASSESSMENTS:**								
***Demographic and socioeconomic information***	X	X						
***General health status***	X			X	X	X	X	X
***Structured clinical interview***	X			X	X	X	X	X
***Pattern identification***	X			X	X	X	X	X
***Psychometric evaluation***								
KSCL95		X		X	X	X	X	X
Hwabyung scales		X		X	X	X	X	X
BDI-II		X		X	X	X	X	X
STAI-KYZ		X		X	X	X	X	X
STAXI		X		X	X	X	X	X
ISI		X		X	X	X	X	X
***Assessment instrument for Korean medicine***								
SPQ		X		X	X	X	X	X
MBI		X		X	X	X	X	X
***Questionnaire for patient perceptions***		X		Δ	Δ	Δ	Δ	Δ
***Short Form 36***		X		X	X	X	X	X
***Heart rate variability***		X		X	X	X	X	X
***Treatment status***		X	X (every month, if possible)	X	X	X	X	X

Δ = optional, KSCL95 = korean-symptom checklist 95, BDI-II = beck depression inventory-II, STAI-KYZ = Spielberger’s state-trait anxiety inventory-form Korean YZ, STAXI = state-trait anger expression inventory, ISI = insomnia severity index, SPQ = sasang personality questionnaire, MBI = mibyeong index, X = mandatory.

#### 2.5.1. Demographic and socioeconomic information.

Age, sex, birth date, body weight, height, body mass index, education, marital status, occupation, religion, income, smoking, and alcohol use will be recorded.

#### 2.5.2. General health status.

We will record information on personal diagnoses and medication use at baseline and the results of physical examination at follow-up.

#### 2.5.3. Structured clinical interview.

We will assess the criteria for DSM disorders, Stagnation syndrome, and Hwabyung and record the results of psychiatric diagnoses and comorbidities.

#### 2.5.4. Pattern identification.

Traditional medicine has its own system that defines and classifies *Pattern* (證). The *International Standard Terminologies on Traditional Medicine* defined a pattern (also called syndrome) as follows: diagnostic conclusion of the pathological changes at a certain stage of a disease, including the location, cause, and nature of the disease, as well as the trend of development; conditions suggesting appropriate treatment; or conditions specific to the individual.^[11]^ The overall process through which a traditional medicine practitioner collects and analyzes clinical data and determines the diagnosis of a pattern is termed “pattern identification” or “syndrome differentiation.”

If the participants are diagnosed with traditional mental disorders (i.e., Stagnation syndrome or Hwabyung), we will identify the following patterns:

- Stagnation syndrome: liver *qi* depression; blood stagnation; liver depression and spleen deficiency; liver-gallbladder dampness-heat; anxiety and depression-damaging spirit; kidney deficiency and liver depression; or dual deficiency of the heart and spleen. These patterns are classified in *Guidelines for Diagnosis and Treatment of Common Internal Diseases in Chinese Medicine Symptoms in Chinese Medicine (ZYYXH/T49-2008*).^[30]^
- Hwabyung: liver *qi* depression; liver fire flaming upward; no interaction between the heart and kidney; dual deficiency of *qi* and blood; or depressed gallbladder with harassing phlegm. These patterns are classified in the *Instrument on Pattern Identifications for Hwabyung*, which was developed and validated by doctors of traditional Korean medicine through literature reviews and expert consensus.^[31]^

#### 2.5.5. Psychometric evaluation.

The primary outcome are the change in the symptom checklist-90-revised (SCL-90-R).^[32]^ The SCL-90-R is a widely used self-report assessment tool to measure psychometric properties and psychiatric symptoms. It comprises 9 dimensions including somatization, obsessive-compulsive, interpersonal sensitivity, depression, anxiety, hostility, phobic anxiety, paranoid ideation, and psychoticism.

We will use the Korean version of the SCL-90-R the Korean version of the checklist-90-revised (KSCL95).^[33]^ In the KSCL95, the validity scale and items reflecting the DSM disorders were added, and they were normalized based on the 2013 Korean demographic distribution. We will analyze each T-score for each domain and sub-domain.

Secondary outcomes are listed below.

##### 2.5.5.1. Hwabyung.

Hwabyung symptoms will be assessed using the two Hwabyung scales. Both scales are self-report questionnaires used for the screening and assessment of Hwabyung patients.

The first was developed in 2008 by a multidisciplinary team comprising psychologists, psychiatrists, and Korean medicine neuropsychiatrists.^[34]^ It is divided into a characteristics scale (16 items) and a symptom scale (15 items), which are rated on a 5-point Likert scale (0–4). In particular, the symptom scale was used as a screening tool for the diagnosis of Hwabyung, with a cutoff score of 29/30.

The other was developed by psychiatrists in 2009,^[35]^ and one of them participated in the development of the former. It comprises 22 symptoms, and each symptom is rated on a 5-point (1–5) or 3-point (1–3) Likert scale, depending on its importance. Total score ranges from 22 to 82 and higher scores indicate higher levels of Hwabyung. The symptoms are divided into 3 clusters (A for Hwabyung-specific symptoms; B for somatic or behavioral symptoms; and C for other related symptoms). If there are 3 or more symptoms with a score of 3 or higher in cluster A and 4 or more symptoms with a score of 3 or higher in cluster B, Hwabyung will be suspected.

##### 2.5.5.2. Depression.

The beck depression inventory-II is a widely used scale to evaluate the severity of depression.^[36–38]^ It consists of 21 symptoms related to the diagnostic criteria for MDD. The Korean version has been validated in healthy university students^[39]^ and in patients with depression.^[40,41]^ Each symptom are graded on a 4-point Likert scale (0–3), with higher scores indicating higher levels of depression.

##### 2.5.5.3. Anxiety.

The state-trait anxiety inventory (STAI) is a self-report questionnaire that measures 2 types of anxiety: state and trait. The original version was STAI-X,^[42]^ which was updated to STAI-Y.^[43]^ We will use STAI-Korean YZ (STAI-KYZ).^[44]^ It consists of 40 questions rated on a 4-point Likert scale (1-4), with higher scores indicating higher levels of anxiety.

##### 2.5.5.4. Anger.

The state-trait anger expression inventory (STAXI) is a self-report questionnaire that measures experience, expression, and control of anger. The first edition consists of 3 scales with a total of 5 subscales (i.e., state anger, trait anger, and anger expression including anger-in, anger-out, and anger-control subscales).^[45]^ Consequently, the second edition of STAXI (STAXI-2) was produced, the subscales were further subdivided, and the number of questions was increased to 57 from 34.^[46]^ However, we will use the 1^st^ edition of the STAXI because it is the only validated version of the STAXI in Korea (STAXI-K).^[47]^ The STAXI-K consists of 34 questions, which are rated using a 4-point Likert scale (1–4); higher scores indicate higher levels of experience and expression.

##### 2.5.5.5. Insomnia.

The insomnia severity index is a brief instrument used to assess insomnia.^[48]^ The insomnia severity index Korean version has been validated in patients with sleep disorders.^[49]^ It consists of 7 questions, each rated on a 5-point Likert scale (0–4), with higher scores indicating higher levels of insomnia.

#### 2.5.6. Assessment instrument for korean medicine.

##### 2.5.6.1. Sasang personality questionnaire.

Sasang constitutional medicine is a typology used in Korean medicine based on Neo-Confucianism. Recently, a research team developed and validated the sasang personality questionnaire, which focused on the yin-yang personality among various characteristics of the Sasang typology and measured cognitive, emotional, and behavioral aspects.^[50]^ The team investigated correlations between the sasang personality questionnaire results and personality traits devised by Cloninger et al.^[51,52]^ We will analyze each percentile score for the 3 domains (cognition, emotionality, and behavior).

##### 2.5.6.2. Mibyeong index.

Korean medicine considers sub-health (“Mibyeong”), a status that requires preventive management. The mibyeong index was designed to assess health status in healthy and subclinical populations.^[53]^ This instrument measures the severity, duration, and resilience of physical symptoms, such as fatigue, pain, sleep disturbance, indigestion, and mental distress, including anxiety, anger, and depression. It was reported that mibyeong index scores was associated with quality of life.^[53,54]^ It consists of 21 questions that are rated on a 7-point Likert scale (1–7). The total score ranges from 21 to 144, but we will analyze the converted score out of 100 using the standard scoring table, with higher scores indicating a worse health status.^[55]^

#### 2.5.7. Questionnaire for patient perceptions.

A semi-structured questionnaire was developed for this study by the researcher (JWK) and reviewed by two Korean medicine neuropsychiatrists (HWS and SYC). It was designed to measure perceived causation and its impact on participants’ distress. The participants will be asked to quantitatively evaluate the impact of various causations as follows: individual’s intrinsic factors, environmental factors, stressful events, reaction or response to stressors, symptoms left as a result, et cetera.

This self-assessment will not be conducted if the criteria for the diagnosis of mental disorders are not met, and the participants subjectively recognize themselves as a state of remission at the follow-up visits.

#### 2.5.8. Short form 36.

Quality of life will be assessed using the short form 36 (SF-36) version 2, also known as the international version.^[56]^ This instrument measures the generic health status and health-related quality of life. It consists of 36 questions encompassing 8 dimensions: physical functioning, bodily pain, social functioning, mental health, general health, vitality, physical role, and emotional role. Using the standard scoring algorithm, scale scores will be converted to a range from 0 to 100, with higher scores indicating better health status.

#### 2.5.9. Heart rate variability.

The characteristics of heart rate variability have been investigated in Hwabyung^[57]^ and depression^[58–60]^ as indicators or biomarkers. We will measure the heart rate variability of the participants repeatedly.

#### 2.5.10. Treatment status.

We will collect information about treatment status, concomitant drugs (CM), and adverse events (AE) at baseline, during treatment (for up to 3 months), and during regular follow-ups. We will investigate CM and AE only if the participants receive treatment in the registry institutions.

At the 1^st^ visit after registration, the clinician will record and collect data based on the participants’ needs, chief complaints, pattern identification, and purposes for using herbal medicine and psychotherapy. During the treatment for up to 3 months, we will collect details of the interventions, the primary outcome KSCL95, CM, and AE every month.

Moreover, in case participants voluntarily complete the consent form for personal information collection and use (separate from the consent form to participate in the registry study), we will obtain the Health Insurance Review & Assessment Service data on medical expenses from 6 months before the participant’s registration to the end of this study.

### 2.6. Sample size

This study focused on descriptive analysis to understand the treatment behavior and medical expenditure status of the study participants. Therefore, it was not necessary to calculate the sample size for hypothesis testing. We plan to recruit 300 participants, considering the budget and research period; however, at least 10 participants will be competitively recruited from each Korean medicine hospital.

### 2.7. Statistical methods

A descriptive analysis was conducted to examine the general characteristics (demographic and socioeconomic information, medical diagnoses, psychiatric diagnoses, etc.) of the participants.

To analyze the effectiveness of Korean medicine treatment for the participants, stratified or corrected analyses of age, sex, and diagnosis will be performed. To evaluate the effect, the difference in the change in psychometric measurements, quality of life measured by SF-36, and quality-adjusted life year will be calculated.

For statistical analysis of this study, SPSS 28 version, Stata MP 17 version, SAS 9.4 version, R 4.1.1 (or the latest version of each program) will be used.

#### 2.7.1. Data managements.

The registry will collect and record participant data using the myTrial solution—a web-based data management system designed to detect missing data or specific errors and display a warning or generate a query. If the inquiry is received, the investigator will revisit the source documents and correct the data. All principal investigators and research coordinators will have access to the final, cleaned datasets.

#### 2.7.2. Monitoring.

A clinical research associate working at the Korean Medicine Clinical Trial Center will monitor source documents at all sites. Source documents were defined as medical charts, associated reports, and records. The clinical research associate will review the source documents to determine the completeness and accuracy of the data recorded in myTrial.

#### 2.7.3. Ethics and dissemination.

The IRB of the Kyung Hee University Korean Medicine Hospital at Gangdong has approved this study (KHNMCOH 2021-04-003). Additionally, this study will receive approval from the IRBs of all participating institutions. Any modifications to the protocol will require a formal amendment to the protocol. All amendments will be approved by the IRB prior to implementation.

Additionally, we will separately obtain the consent form for personal information collection and use to analyze Health Insurance Review & Assessment Service data.

All study-related information will be stored securely in locked file cabinets in areas with limited access at the study site.

The results of this study will be submitted for publication in a peer-reviewed journal.

## 3. Discussion

The KMental study will register up to 300 patients with mental health issues visiting neuropsychiatric outpatients in Korean medicine hospitals. Korean medical neuropsychiatrists actively manage patients with mental disorders in Korea, but these facts are not well-known. Furthermore, in our experience, although many Koreans suffer psychiatric distress as a form of Stagnation syndrome or Hwabyung, relevant research and observational studies are lacking. This registry aims to narrow the evidence gap and has the following strengths:

First, we will describe the general characteristics of patients using Korean medicine for mental health. In general, women, older age groups, worse health conditions, presence of chronic disease, a degree of knowledge about Korean medicine, and a view about herbal medicine safety are related to the use of Korean medicine.^[61]^ Similarly, a survey conducted in Europe reported that middle-aged women (35–74 years) with higher education and higher income were likely to use traditional Asian medicine.^[62]^ However, to date, these determinants have not been examined in clinical populations with mental health disorders. We expect to gain a better understanding of psychiatric patients using Korean medicine in this study.

Second, although people worldwide use complementary and alternative medicine (CAM), including traditional Asian medicine, to treat depression and anxiety,^[63–65]^ most surveys have not investigated the details of interventions and treatment frequency/duration because they enabled the use of hospital data and collected data based on participants’ memory. We will use a standardized form to record the details of Korean medicine interventions based on medical records. Thus, we can understand how Korean medical neuropsychiatrists manage their patients and what core interventions are used in mental health.

Third, there are concerns that the use of CAM or traditional medicine results in undesirable delays in the diagnosis and treatment of chronic illnesses (e.g., cancer^[66,67]^ and rheumatic arthritis^[68]^). The American Psychiatric Association Task Force also stated that spreading CAM therapies could delay the well-established conventional treatment of depression.^[69]^ However, at the same time, the publication of meta-analyses supporting the use of CAM and traditional medicine for mental disorders has increased. Although certain conclusions were not based on high-quality evidence, recent systematic reviews have suggested that herbal medicine and acupuncture interventions used in traditional Asian medicine are effective and safe for treating depression immediately after treatment.^[70,71]^ Our study may provide observational data on the long-term effects and safety issues of Korean medicine based on follow-ups for at least 4 years.

Finally, we provided a standard operating procedure and established electronic case report forms. We attempted to advance our data management strategy.

However, this registry has a few limitations. First, we will only include Korean medicine hospitals as registry institutions, even though there are many primary clinics for Korean medicine. Additionally, we will not include all outpatients but only those who agree to participate in this study. Thus, we have established a system that registers a small proportion of the total population. We hope that further studies will include various healthcare settings and more participants. Second, our inclusion/exclusion criteria were not strict and heterogeneous populations were registered. We allowed loose criteria to identify frequent complaints and comorbidities with various mental disorders, including Stagnation syndrome and Hwabyung. Based on our results, elaborate observational and experimental studies should be planned.

In conclusion, KMental aims to recruit 300 patients from more than 8 Korean medicine hospitals in Korea from March 2022. Our registry will provide information on people who use Korean medicine for mental health, including comprehensive demographic and socioeconomic characteristics, presentations and severity of their symptoms, and patterns of medical use, and information about the details and long-term results of Korean medicine. Furthermore, KMental will facilitate research collaborations and further studies on traditional mental disorders such as Stagnation syndrome and Hwabyung.

## Acknowledgments

The authors thank Eunhee Kim of the Korean Medicine Clinical Trial Center, Kyung Hee University Korean Medicine Hospital, and Saebom Hyun of Kyung Hee University for their advice on the study design.

## Author contributions

**Conceptualization:** Hyo-Weon Suh, Youme Ko, Jong Woo Kim, Bo-Hyoung Jang.

**Funding acquisition:** Jong Woo Kim.

**Methodology:** Hyo-Weon Suh, Youme Ko, Seunghwan Moon, Jong Woo Kim, Sun-Yong Chung, Sunggyu Hong, Hyun Woo Lee, Bo-Hyoung Jang.

**Project administration:** Bo-Hyoung Jang.

**Writing – original draft:** Hyo-Weon Suh.

**Writing – review & editing:** Ching-Wen Huang, Bo-Hyoung Jang.
